# Role of Re-Resection in Non–Muscle-Invasive Bladder Cancer

**DOI:** 10.1100/tsw.2011.29

**Published:** 2011-02-03

**Authors:** Harry W. Herr

**Affiliations:** Memorial Sloan-Kettering Cancer Center, New York, USA

**Keywords:** transurethral resection, restaging, bladder tumors

## Abstract

Restaging, or second transurethral resection (TUR), is essential to successful management of high-risk, non–muscle-invasive bladder cancer. Here we review the relevant literature documenting the role of restaging TUR. Cohort and randomized studies show that restaging TUR detects more tumors than initial TUR, improves clinical staging, and reduces the frequency of early tumor recurrences. Our conclusions show thatrestaging TUR improves the outcomes of high-risk, non–muscle-invasive bladder neoplasms.

## INTRODUCTION

Transurethral resection (TUR) is the essential surgical procedure used to diagnose, stage, and treat non–muscle-invasive bladder cancer (NMIBC). TUR of bladder tumors is a diagnostic, prognostic, and therapeutic operation.TUR aims to provide adequate specimen material in order to determine the histologic type and grade of bladder tumors, to determine the presence and depth of tumor invasion, and to remove visible and microscopic superficial and invasive tumors.The findings are used to direct further therapy, dictate follow-up schedules, and indicate prognosis[1].

Although TUR is a frequently performed operation familiar to urologists, it is a stochastic procedure subject to unquantifiable surgeon-, tumor-, and pathologically related variables.As a result, its diagnostic and therapeutic purposes are not always achieved.To overcome these limitations, a second, or re-resection,performed 2–6 weeks after an initial TUR has been incorporated into routine clinical practice, especially for high-risk tumors[2].

## RATIONALE FOR RE-RESECTION OF BLADDER TUMORS

Successful management of NMIBC is driven by two overarching principles, one dependent on the other.First, all visible papillary tumors, especially invasive T1 lesions, should be completely resected.Second, intravesical therapy is most effective when used against minimal residual disease.For example, intravesicalbacillus Calmette-Guerin (BCG) is used to eradicate carcinoma *in situ*, not to treat T1 tumors.Despite these caveats, residual tumor is frequently found on a contemporary second TUR, and persistent tumors are commonly found at the 3-month cystoscopy after diagnosis, often at the same sites of disease.A third variable that affects the outcome of NMIBC is pathologic evaluation of tumor specimens.Although pathologists may differ in their interpretation, they can only assess what they are given by the urologist.As an example, one study found that muscularispropria was missing in up to 51% of TUR specimens[3].Furthermore, tumors are mostoften submitted in multiple pieces, from which pathologists are asked to reconstruct the whole tumor type, configuration, and extent.It seems reasonable that the more specimens that are submitted, the greater the likelihood a pathologist will correctly define a tumor(s).These facts argue that a restaging TUR may better stage NMIBC as well as provide better local control, rather than relying on the initial TUR alone.

## DETECTION OF RESIDUAL TUMOR AND REDUCING STAGING ERRORS

The rate of residual tumor detected by a second TUR varies between 27 and 78%[4].Disease persists in 27–72% of Ta and 33–78% of T1 tumors.Multiple- and high-grade or -stage tumors are at greater risk for incomplete resection.Table 1 shows our updated results in 1,312 patients with NMIBC referred by outside urologists in whom we performed a second contemporary TUR[1].A significant proportion (74%) was found on the second TUR to have residual tumor and only 26% of cases had no tumor found in the bladder.Restaging TUR was not helpful for low-grade papillary tumors.However, for patients with high-grade lesions, half the Ta tumors had residual disease and 15% were upstaged; for T1 tumors, 48% had persistent NMIBC and 30% were upstaged to muscle invasion.The second TUR staged papillary tumors more accurately, while providing better local treatment of multipleprimary lesions at the same time.

**TABLE 1**
**Restaging pTa and pT1 Bladder Tumors**

| Tumor Type | No. Patients | Percent Pathology on Restaging TUR | | | | |
|---|---|---|---|---|---|---|
| | | pT0 | pTaLG | pTaHG/CIS | pT1 | pT2 |
| pTaLG | 215 | 49 | 46 | 5 | 0 | 0 |
| pTaHG | 396 | 35 | 0 | 50 | 10 | 5 |
| pT1 | 701 | 22 | 0 | 23 | 25 | 30 |
| Muscle | 421 | 25 | 0 | 31 | 29 | 15 |
| No muscle | 280 | 20 | 0 | 15 | 20 | 45 |

LG: low-grade; HG: high-grade papillary tumors.

## RESTAGING TUR HELPS SELECT PATIENTS FOR CYSTECTOMY VS.INTRAVESICAL THERAPY

Despite judicious management, NMIBC progresses to muscleinvasion in 20–30% cases, usually within 5 years.Once that occurs, survival is worse than if cystectomy had been performed before muscleinvasion[5].Fig. 1 shows progression-free survival of 710 patients with NMIBC who received BCG therapy after an initial (left) or restaged (right) TUR, stratified by stage and grade.After an initial TUR, all tumor categories appear to be appropriate candidates for intravesicaltreatments.However, the majority of patients with residual T1 disease on second TUR eventually progressed (regardless of their original pathology), including many who responded initially to BCG therapy[6].We advise patients who have T1 NMIBC on a second TUR to undergo an immediate cystectomy[7].Patients with no tumor or non-T1 disease on restaging TUR are candidates for bladder-sparing treatments.This has been confirmed in a recent study in which patients having no tumor on repeat resection had fewer recurrences, longer times to tumor recurrence, and less progression than patients who underwent a single TUR[8].

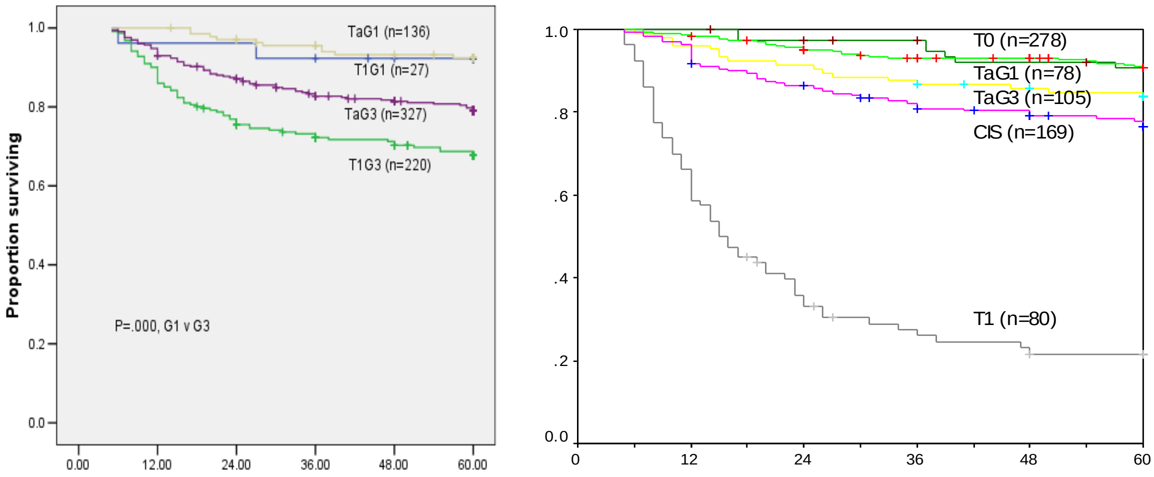

**FIGURE 1.**Progression-free survival of 710 NMIBC patients, according to stage and grade, after first (left) or second (right) TUR.

## RESTAGING TUR IMPROVES OUTCOMES OF NMIBC

A 5-year observational study in 124 patients showed that 63% undergoing a second TUR had tumor-free bladders compared to 40% of patients after one TUR[9].Progression to muscleinvasion occurred in only two (3%) patients after restaging TUR.A second therapeutic TUR also appeared to improve the short-term response to BCG therapy[10].In a prospective, nonrandomized, phase II study of 340 cases, 59% of the patients after one TUR had recurrence by 12 months compared to 16% after two TURs; after 5-years follow-up, 32% of patients had progressed after one TUR vs. 7% after two TURs.Fig. 2 shows that overall recurrence-free and progression-free survival was significantly better with BCG therapy after two vs. one TUR.

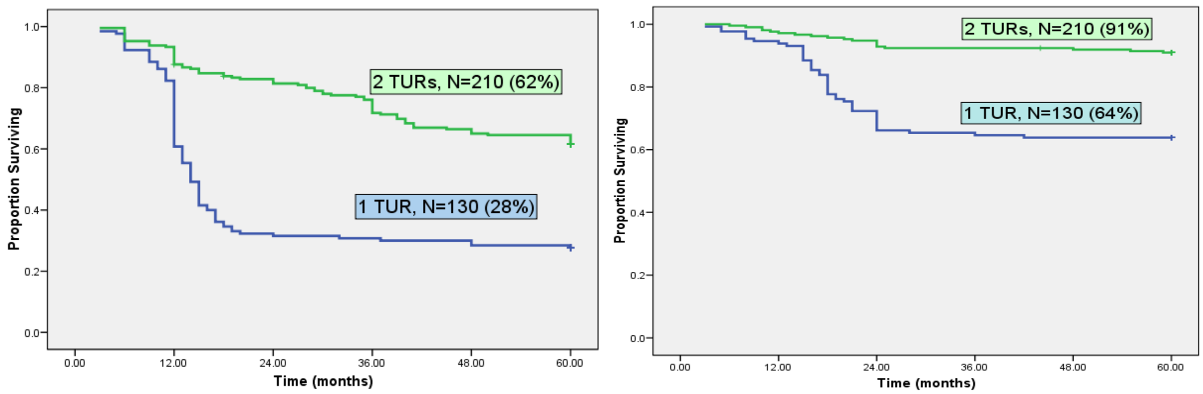

**FIGURE 2.** Recurrence-free (left) and progression-free (right) survival in 340 NMIBC patients treated with BCG after one vs. two TURs.

In a prospective, randomized trial involving 210 patients, Divrik et al.[11] showed that 40% recurred after two TURs compared with 71% after one TUR.Fig. 3 shows longer recurrence-free (47 months) and progression-free (73 months)survival times after the second TUR rather than no second TUR (12 and 53 months, respectively).Overall survival in both groups was similar.However, only 2% of patients died of urothelial cancer after undergoing two TURs compared with 11% after one TUR[11].

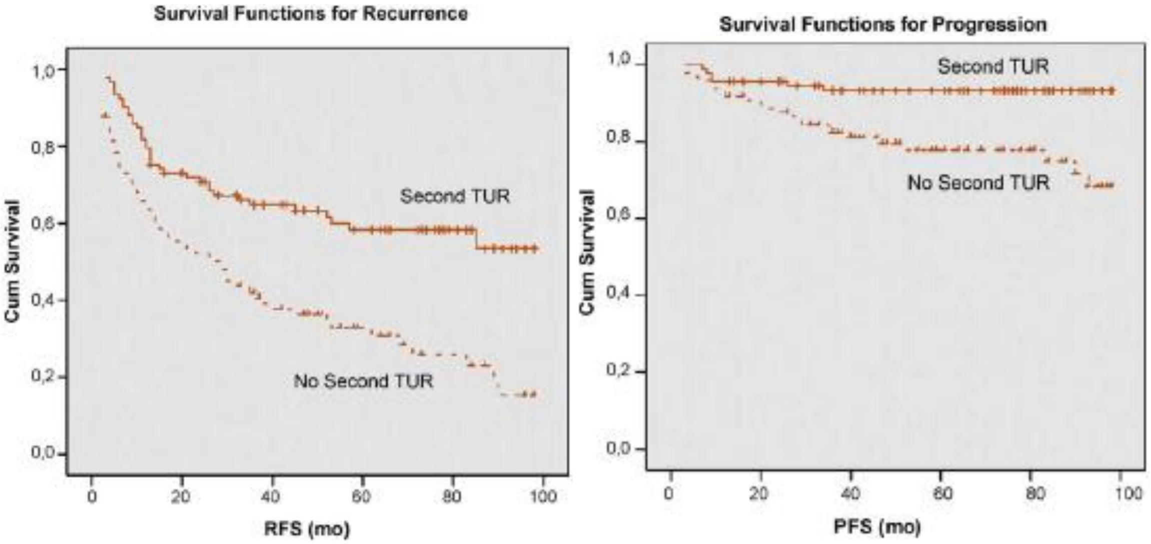

**FIGURE 3.** Recurrence-free and progression-free survival in pT1 bladder cancer patients after one vs. second TUR[11].

The methodology of this study has been criticized because nearly 8% of patients who upstaged to muscleinvasion by the second TUR were excluded from analysis.However, this ignores the fact that restaging TUR resulted in better patient selection, leading to better outcomes for true T1 disease, exactly what a good procedure is designed to do[12].Similarly, although we found that disease-specific survival for T1 bladder cancer was unaltered by the number of TURs, this was because the second TUR identified and selected the highest-risk patients for immediate cystectomy, whereas others received and responded to BCG therapy[13].The collective experience is a strong argument for, rather than against, a restaging TUR.

## QUALITY OF TUR

Analysis of seven randomized trials showed substantial variation in early recurrence rates among different institutions.The frequency of 3-month recurrences ranged from 0 to 46%, owing to the quality of TUR performed by different surgeons[14]. Even experienced urologists have a high percentage of persistent carcinoma after performing the initial TUR.Of 214 patients undergoing restaging TUR for TaT1 tumors, carcinoma was present in 37% treated by senior urologists and 26% treated by urologists in training[15].In an earlier study, deep resection of the bladder wall underlying the tumor and surrounding areas detected more cancer in 13% of Ta and 35% of T1 tumors[16].Likewise, we performed the first and second TUR in 71 T1 patients and found that 25% had residual lamina propriainvasion[17].

The question is:Why is there a high rate of persistent tumor after the first TUR for NMIBC, or put another way, why can’t even experienced urologists do it right the first time?The answer lies in the technical difficulties of the procedure itself and in the nature of bladder tumors.Visibly complete TUR of multiple papillary lesions, especially T1 tumors associated with carcinoma *in situ*, is often difficult the first time.Tumors may be overlooked if extensive or involve sometimes difficult to reach regions of the bladder, such as dome, anterior wall, bladder neck, or urethra.Tumor spread at the margins or invading lamina propria isnot always seen at cystoscopy and is often more extensive than the surface appearance of the tumor suggests.As the resection proceeds, vision often becomes obscured, owing to mucosal edema, bladder spasms, and bleeding, making it increasingly difficult to differentiate benign from tumor-bearing mucosa, and to obtain clear negative surgical margins.TUR is a random, partially blind, and technically difficult procedure that, combined with uncertainty of tumor growth, conspires to limit the urologist's ability to always perform a reliably complete TUR!We can teach ourselves and others to perform high-quality TURs[18], however,that alone will not completely negate the value or need for a contemporary second TUR for most NMIBC[1].Perhaps new optical enhancement methodsunder investigation, such as fluorescence cystoscopy and narrow band imaging, will help to improve the performance of the initial TUR.However, they will not solve the problem of understaging.

## WHO SHOULD HAVE SECOND TUR?

A second TUR is recommended for any high-grade NMIBC detected at the initial TUR[2].A second TUR achieves better local control, improves response to intravesical therapy, reduces understaging, and often leads to changes in treatment resulting in better outcomes, defining it as a diagnostic, therapeutic, prognostic, and predictive procedure.Most authors recommend resection 2–6 weeks after the initial TUR, which should include a thorough TUR of the primary tumor site, any overlooked tumors, and, in my opinion, all overt or suspected areas of carcinoma *in situ*.In the majority of cases, results are better when all tumors have been eradicated before subsequent intravesicaltreatments.

On the other hand, NMIBC represents a heterogeneous spectrum of diseases and not all need or benefit from a second TUR.For example, another TUR is not helpful, nor does it change management for low-grade papillary tumors.In addition, we found that low-grade T1 tumors, albeit rare, are easily controlled by the first TUR[19].Others have questioned whether a second TUR is necessary after a well-performed resection of high-grade T1 that displays limited lamina propria invasion (T1a) and when BCG treatment is planned[20].These latter exceptions are reasonable, but only if the urologist is confident in his/her ability to completely resect all tumors (seen and unseen), and knows the capability of the pathologist.Restaging, or second, TUR is required for multiple tumors, high-grade tumors, the majority of T1 tumors, incompletely resected tumors, and if no muscle is identified in submitted tumor specimens.

## CONCLUSIONS

Second, or restaging, TUR of NMIBC achieves optimal local control by removing residual tumors, improves staging accuracy, provides additional histologic material favoring more accurate diagnosis, leads to changes in treatment with improved outcomes, facilitates response to intravesical therapy, and provides important prognostic information.Restaging TUR is a diagnostic, therapeutic, prognostic, and predictive procedure, essential to successful management of high-risk NMIBC.

## References

1. Herr, H.W. and Donat, S.M. (2008) Quality control in transurethral resection of bladder tumors. *BJU Int.***102,** 1242–1246.
2. Bubjuk, M., Oosterlinck, W., Sylvester, R., et al. (2008) EAU guidelines on non-muscle-invasive urothelial carcinoma of the bladder. *Eur. Urol.***54,** 303–314.
3. Maruniak, N.A. et al. (2002) Accurate pathologic staging of urothelial neoplasms requires better cystoscopic sampling. *J. Urol*. **167,** 2404–2407.

4. Babjuk, M. (2010) Second resection for non-muscle-invasive bladder carcinoma: current role and future perspectives. *Eur. Urol*. **58,** 191–192.
5. Raj, G. et al. (2007) Treatment paradigm shift may improve survival of patients with high risk superficial bladder cancer. *J. Urol.***177,** 1283–6.
6. Herr, H.W. et al. (2007) Can restaging transurethral resection of T1 bladder cancer select patients for immediate cystectomy? *J. Urol*. **177,** 75–79.
7. Dalbagni, G. et al. (2009) Clinical outcome in a contemporary series of restaged patients with T1 bladder cancer. *Eur. Urol.***56,** 903–910.
8. Guevara, A. et al. (2010) The role of tumor-free status in repeat resection before intravesical BCG for high grade Ta,T1 and CIS bladder cancer. *J. Urol*. **183,** 2161–2164.
9. Grimm, M.-C. et al. (2003) Effect of routine repeat transurethral resection for superficial bladder cancer: a long-term observational study. *J. Urol*. **170,** 433–7.
10. Herr, H.W. (2005) Restaging TUR of high risk superficial bladder cancer improves the initial response to BCG therapy. *J. Urol*. **174,** 2134–2137.
11. Divrik, R.T. et al. (2010) Impact of routine second TUR on the long-term outcome of patients with newly diagnosed pT1 urothelial carcinoma with respect to recurrence, progression rate, and disease-specific survival: a prospective randomized clinical trial. *Eur. Urol.***58,** 185–190.
12. Novara, G. et al. (2010) Does routine second TUR affect long-term outcome of patients with T1 bladder cancer? Why a flawed randomized controlled trial cannot address the issue. *Eur. Urol.***58,** 193–194.
13. Dalbagni, G. et al. (2010) Variability of treatment selection among surgeons for patients with cT1 urothelial carcinoma. *BJU Int.***106**, 1502–1507.
14. Brausi, M.A. et al. (2002) Variability in the recurrence rate at the first follow-up cystoscopy after TUR in stage TaT1 transitional cell carcinoma of the bladder: a combined analysis of seven EORTC studies. *Eur. Urol.***41,** 523–531.
15. Zurkirchen, M.A. et al. (2004) Second TUR of superficial transitional cell carcinoma of the bladder: a must even for experienced urologists. *Urol. Int*. **72,** 99–102.
16. Koloszy, Z. (1991) Histopathological “self control” in transurethral resection of bladder tumors. *BJU Int.***67,** 162–164.
17. Dalbagni, G. et al. (2002) Impact of a second transurethral resection on staging of T1 bladder cancer. *Urology***60,** 822–825.
18. Pycha, A. et al. (2007) How to teach the teacher to teach the TUR-B. *Int. J. Surg*. **5,** 81–85.
19. Herr, H.W. et al. (2006) A re-staging transurethral resection predicts early progression of superficial bladder cancer. *BJU Int*. **97,** 1194–1198.
20. Orsola, A. et al. (2009) Letter to editor. *Eur. Urol.***55,** e15–16.
